# SeqPurge: highly-sensitive adapter trimming for paired-end NGS data

**DOI:** 10.1186/s12859-016-1069-7

**Published:** 2016-05-10

**Authors:** Marc Sturm, Christopher Schroeder, Peter Bauer

**Affiliations:** Institute of Medical Genetics and Applied Genomics, University Hospital Tübingen, Tübingen, Germany

## Abstract

**Background:**

Trimming of adapter sequences from short read data is a common preprocessing step during NGS data analysis. When performing paired-end sequencing, the overlap between forward and reverse read can be used to identify excess adapter sequences. This is exploited by several previously published adapter trimming tools. However, our evaluation on amplicon-based data shows that most of the current tools are not able to remove all adapter sequences and that adapter contamination may even lead to spurious variant calls.

**Results:**

Here we present SeqPurge (https://github.com/imgag/ngs-bits), a highly-sensitive adapter trimmer that uses a probabilistic approach to detect the overlap between forward and reverse reads of Illumina sequencing data. SeqPurge can detect very short adapter sequences, even if only one base long. Compared to other adapter trimmers specifically designed for paired-end data, we found that SeqPurge achieves a higher sensitivity. The number of remaining adapter bases after trimming is reduced by up to 90 %, depending on the compared tool. In simulations with different error rates, we found that SeqPurge is also the most error-tolerant adapter trimmer in the comparison.

**Conclusion:**

SeqPurge achieves a very high sensitivity and a high error-tolerance, combined with a specificity and runtime that are comparable to other state-of-the-art adapter trimmers. The very good adapter trimming performance, complemented with additional features such as quality-based trimming and basic quality control, makes SeqPurge an excellent choice for the pre-processing of paired-end NGS data.

**Electronic supplementary material:**

The online version of this article (doi:10.1186/s12859-016-1069-7) contains supplementary material, which is available to authorized users.

## Background

Adapter contamination is a common problem of short-read sequencing. It arises from fragments of the sequencing library that are shorter than the read length itself. In that case, a ‘read through’ into the sequencing adapters occurs after sequencing the actual insert of interest. These adapter sequences often disturb downstream analysis of the data, i.e. read mapping and variant calling, or *de-novo* assembly of reads.

Thus, trimming adapter sequences is a common preprocessing step in most NGS data analysis pipelines. For amplicon-based sequencing approaches, which are widely used in clinical diagnostics, sensitive adapter trimming is of special importance. Recurrent untrimmed adapters at the same genomic position can lead to spurious variant calls. Shotgun sequencing approaches with random distribution of reads over the target region are more robust towards adapter contamination. The random read distribution reduces the probability of spurious variant calls for most variant calling applications. However, when calling variants with low allele frequencies, e.g. somatic variants or mosaic variants, adapter contamination can still lead to spurious variant calls. Therefore, adapter trimming is an essential step for shotgun data as well.

**Fig. 1:**
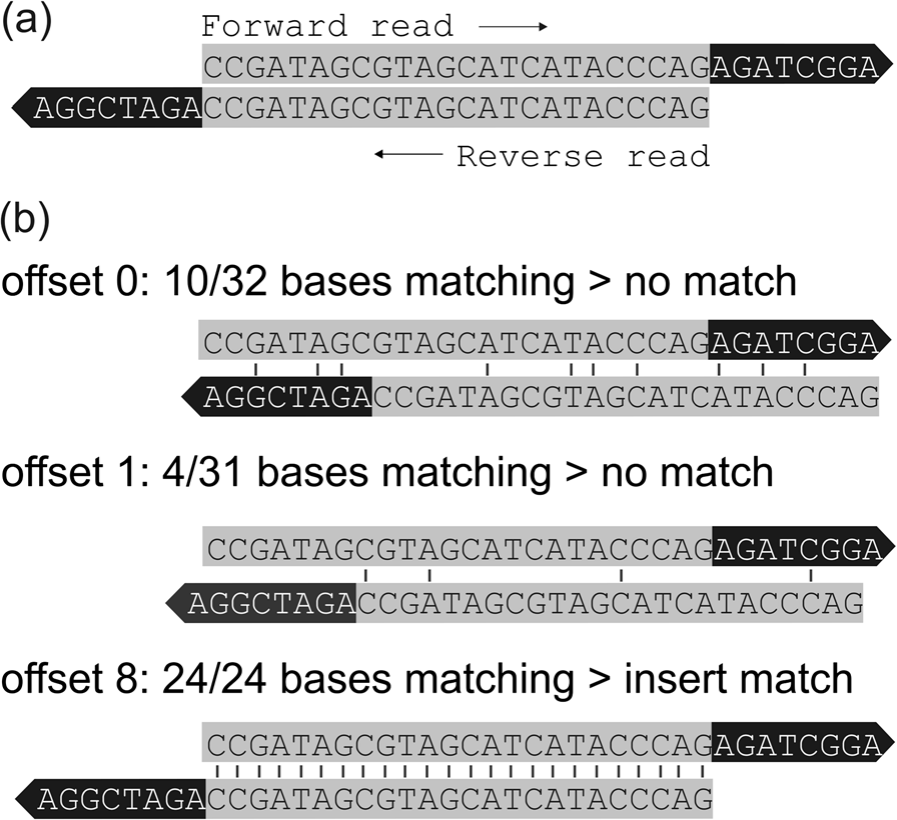
Read layout with adapter contamination (**a**) and insert match algorithm examples with different offsets (**b**). Inserts are colored grey, adapter remains are colored black. Reverse reads are displayed with reverse-complementary sequence to facilitate visual comparison of sequences

Adapter trimming algorithms typically try to find the adapter sequences in the reads using semi-global alignment or similar techniques. Jiang et al. [5] give a detailed overview of the algorithms used in various tools. Algorithms designed specifically to handle paired-end data often take a different approach than those for single-end data. Adapter contamination in paired-end data means that the insert, i.e. the fragment of interest, was completely sequenced in both reads (see Fig. 1a). Most tools that are designed for paired end data use the match of insert sequences instead of searching for adapter sequences. This approach has the big advantage that it can trim very short adapter residues, down to one base length. We will term this the *insert match approach.* The alternative approach, where adapters are detected based on their sequence only, is called *adapter match approach*.

While analyzing NGS data generated with the amplicon-based HaloPlex enrichment (Agilent, Santa Clara CA), we found that adapter contamination was not fully removed by any of the adapter trimming tools we applied, even with optimized parameters [1, 8, 10]. Thus, we developed SeqPurge, a very sensitive paired-end adapter trimmer that is based on a probabilistic approach.

## Implementation

Before going into the algorithm details, we will briefly define the problem more formally: Given forward/reverse adapter sequences and the forward/reverse read produced from one DNA fragment, remove all bases from the reads that stem from a read-through into the adapters, if present. Those bases that must not be trimmed because they stem from the actual sequence of interest are called *insert* in the following text.

### Calculation of non-random match probability

As mentioned in the introduction, our algorithm is not based on sequence alignment. It uses a rather simple probabilistic approach. Given two DNA/RNA sequences of length *n*, we count the number of matching bases *k* between the sequences. Given the probability *p* of a single base match, we calculate the probability *P* to observe *k* or more matching bases in sequences of length *n* using the binomial distribution:

$$ P={\displaystyle \sum_{i=k}^n}\frac{n!}{i!\left(n-i\right)!}\ {p}^i{\left(1-p\right)}^{n-i} $$

We call two sequences a (non-random) match, if *P* is less than a given threshold. This threshold is the main parameter that balances sensitivity/specificity of our algorithm.

Using this simple approach is possible because modelling indels is not necessary - an insertion/deletion in the insert is present in both read directions. We use a fixed match probability *p* of 0.25 for all bases, i.e. we assume that all four bases occur at the same rate. Based on our performance evaluations, this simplification causes no major problems.

### Algorithm description

The primary design goal of SeqPurge was to achieve a very high sensitivity while maintaining a state-of-the-art specificity. In general, the insert match approach is very sensitive and thus is the best approach for paired-end reads. However, certain sequence motives and unbalanced base content can make sequencing difficult in one read direction or even in both read directions. In these difficult sequences, the adapter match approach can perform better than the insert match approach. Thus, we combine the two approaches to increase the sensitivity of SeqPurge.

First, we try to find an insert match between forward read and the reverse-complement of the reverse read. To detect an insert match, each possible offset between both reads is tested for a match (see Fig. 1b). If we find a match, the adapters are trimmed and the insert remains. If we find several matches, we select the best offset, i.e. the one with the lowest probability of being random. Several matches occur primarily in reads of regions with simple repeats. To prevent overtrimming because of false-positive matches in simple repeat regions, we also require a match between the previously defined adapter sequence and the sequence flanking the putative insert. This additional adapter match is required in only one out of the two reads, which makes the algorithm more robust towards bad read quality in one read direction.

If no insert match was found, we check for adapter matches in the forward and reverse read separately. Again, each possible offset is tested for an adapter sequence match. If an adapter match is detected, the read is trimmed starting at the offset position. If only one of the two reads has an adapter match, the other read is trimmed at the offset as well, because a ‘read through’ is always symmetrical. The rationale is again that one read could have bad quality due to sequencing problems.

### Theoretical runtime and runtime optimizations

The theoretical runtime of the algorithm is *nm*^*2*^ where *n* is the number of reads and *m* is the read length. We implemented several optimizations to reduce the runtime in practice.

Because we need to calculate the match probability *m*^*2*^ times for each read pair and three faculty values are required for the calculation, the faculty values are pre-calculated and stored in a hash for fast lookup.

To avoid a large part of the match probability calculations, we added a minimum matching bases parameter (default is 80 %). Once the maximum allowed number of mismatches of a comparison is exceeded, the rest of the comparison can be skipped. This reduces the overall runtime by up to 75 %.

Reading the data from file and processing the data in the same thread can slow down the analysis considerably when file I/O is slow. Thus, we pre-fetch reads in an I/O thread and use *n* additional threads for data analysis (*n* = 1 by default). This strategy of course increases the memory usage slightly.

We are currently evaluating the possible speedup when using four bit-arrays instead of characters to store DNA sequences. This approach is also used by Skewer [5], the fastest tool in our comparison.

## Results

For our benchmark we selected the best tools for paired-end reads from [5]: Skewer [5], AdapterRemoval [8], Trimmomatic [1], SeqPrep (https://github.com/jstjohn/SeqPrep) and FlexBar [2]. Additionally, we benchmarked the recently published tool PEAT [7], which was not part of the comparison by Jiang and colleagues.

### Benchmark datasets

For the main performance comparison, we used real-world data to make the benchmark as realistic as possible. We created a benchmark dataset by sequencing the NIST reference sample NA12878 [13]: (1) A library was created from the NA12878 sample using a HaloPlex custom panel containing the exons of 71 genes (247,305 bases in total) related to hereditary breast and ovarian cancer; (2) the library was sequenced on an Illumina MiSeq (Illumina Inc, San Diego, CA) resulting in about half a million paired-end reads of 158 bp length. An amplicon dataset was used for the benchmark, because the effect of incomplete adapter trimming can even be seen in variant lists. Generally, an adapter trimmer that performs well on amplicon data will perform equally well on shotgun data.

For a second benchmark, we simulated paired-end data. Five million read pairs for the coding region of the genome (CCDS) were created with varying error rates of 0 to 4 %. The 100 bp read pairs were created based on a theoretical library with mean insert size of 100 bp and a standard deviation of 50 bp. This results in a dataset where 50 % of the reads contain adapter contamination and need to be trimmed. The reads were simulated using the PERsim (see Availability of data and materials).

### Benchmark metrics

We compare the adapter trimming performance based on metrics of raw reads, mapped reads and variants called. For each of the three steps, different metrics are used.

For raw reads, the number of bases remaining after trimming is counted. Additionally, we report the number of remaining adapter 20-mers from the beginning of the adapters. This crude metric already gives a good impression of the sensitivity. After perfect adapter trimming no adapter 20-mer should be found in trimmed data.

For mapped reads, the first metric is the number of properly paired reads with a mapping quality of 40 or higher. The high mapping quality cutoff of 40 was chosen to remove low-accuracy alignments. Removing low-accuracy alignments is important because alignments are the basis to calculate the metrics of overtrimmed and undertrimmed bases: The start positions of the forward and reverse read indicate the start and end position of the insert, respectively (see Fig. 1a). These metrics depend on the mapping software and its parameters. For the real data evaluation, we assume that all alignments are correct and that the Phred mapping quality is well-calibrated. For the simulated data evaluation, no mapping is required and the correct trimming is known a priori.

For variants, the most basic metric is the overall number of variants called. This simple metric can be used to identify adapter trimmers that cannot trim short adapter fragments. Remaining adapter sequences result in higher variant counts. The more significant metrics are the number of uncalled true-positives and called false-positives. To determine these, all variants with genotype calls differing between tools were manually inspected.

### Benchmark results on real data

For this benchmark, we performed adapter trimming with the respective trimming tool, mapping with BWA 0.7.5 [6] and variant calling with freebayes 0.9.20-16 [3].

All adapter trimmers were configured similarly to make the results comparable: one thread was used for trimming; reads smaller than 15 bases after trimming were discarded; no trimming by base quality or no-call stretches (i.e. no base could be called) was performed. For algorithm-specific parameters of the adapter trimmers default values were used. Mapping was performed using the BWA “mem” algorithm with default parameters. Variant calling was performed on bases with a Phred base quality of 20 or higher only.

Table 1 summarizes the raw data metrics after trimming. The first line shows that more than 400,000 exact adapter 20-mers are present in the input data. All adapter trimmers significantly reduce the number of adapters, but only SeqPurge and SeqPrep can remove all adapters without any parameter optimization. Trimmomatic and FlexBar remove nearly all adapter 20-mers. AdapterRemoval, Skewer and PEAT leave a significant amount of adapter sequences in the reads.

**Table 1:** Adapter trimming benchmark results on real data (raw reads)

	Adapters left	Bases left
no trimming	414254	168509790
SeqPurge 0.1-270	0	142570393
AdapterRemoval 1.5.4	**1450**	142864048
Flexbar 2.5	7	142323263
PEAT 1.2.2	**24298**	**146433832**
SeqPrep 1.2	0	**142069127**
Skewer 0.1.123	**425**	142664746
Trimmomatic 0.32	5	142717579

The number of adapter 20-mers and the number of bases left in the raw read data after adapter trimming. The most notable entries are highlighted (bold)

In terms of bases left after trimming, most tools produce roughly 142.5 million bases. SeqPrep removes significantly more bases. This will be discussed in the next section together with the number of properly-paired reads. PEAT removes significantly less bases, because PEAT keeps reads with an insert size of zero, i.e. adapter-dimers with no insert.

Table 2 shows the performance metrics after mapping. The properly-paired read count for most tools lies between 1021224 and 1021323 reads. Interestingly, the properly-paired read count for untrimmed reads is higher. Intuitively one would argue that adapter trimming should increase the number of mappable reads. However, this contradiction can be explained. Untrimmed adapters can make reads with very short inserts uniquely mappable which would not be uniquely mappable without adapter. Even reads without any insert can be mappable when allowing soft-clipping of reads during the mapping, which BWA does (see Additional file 1: Figure S1 for an example). Only SeqPrep and PEAT are outliers in terms of properly-paired read count. For SeqPrep the properly-paired read count is lower because it completely removes about 4000 reads no other trimmer removes. Manual inspection revealed that most of these reads are of high quality and are mappable as a proper pair. It remains unclear why SeqPrep removes these reads (see Additional file 1: Figure S3 and Figure S4 for examples). PEAT cannot remove reads with no insert. These untrimmed reads lead to a seemingly higher properly paired read count.

**Table 2:** Adapter trimming benchmark results on real data (mapping)

	Reads paired	Bases overtrimmed	Bases undertrimmed
no trimming	**1022388**	0	21918793
SeqPurge 0.1-270	1021315	1736	33650
AdapterRemoval 1.5.4	1021290	25	**223997**
Flexbar 2.5	1021224	**4539534**	62901
PEAT 1.2.2	**1022901**	**69296**	**354531**
SeqPrep 1.2	**1018828**	53	34949
Skewer 0.1.123	1021323	238	**95279**
Trimmomatic 0.32	1021316	1580	**190062**

Benchmark results after mapping: properly-paired reads, erroneously trimmed insert bases, untrimmed adapter bases. The most notable entries are highlighted (bold)

When looking at overtrimmed and undertrimmed bases, only SeqPurge and SeqPrep show balanced sensitivity (i.e. a low number of undertrimmed bases) and specificity (i.e. a low number of overtrimmed bases). FlexBar and PEAT have a low specificity. AdapterRemoval, PEAT, Skewer and Trimmomatic have a low sensitivity.

Table 3 shows the benchmark results of the variant calling step. 155 variants were called after trimming with each of the adapter trimming tools. These consensus variants were considered true-positives and, thus, were not further evaluated. All variants with diverging calls were manually inspected using IGV [12].

**Table 3:** Adapter trimming benchmark results on real data (variant calling)

	Variants	Uncalled TPs	Called FPs
no trimming	**178**	**1**	**24**
SeqPurge 0.1-270	155	0	0
AdapterRemoval 1.5.4	155	0	0
Flexbar 2.5	**174**	0	**19**
PEAT 1.2.2	155	0	0
SeqPrep 1.2	155	0	0
Skewer 0.1.123	155	0	0
Trimmomatic 0.32	**178**	0	**23**

Benchmark results after variant calling: overall variant count, number of true-positive variants that were not called, number of false-positive variants that were called. The most notable entries are highlighted (bold)

Without adapter trimming, 24 spurious variants were called. They were all caused by short adapter remains in mapped reads. The high number of spurious variant calls underlines the importance of highly sensitive adapter trimming for amplicon-based sequencing data. Using FlexBar and Trimmomatic 19 and 23 false-positive variants were called, respectively, because they failed to trim short adapter remains. FlexBar trims adapter sequences longer than two bases only. Trimmomatic trims adapter sequences longer than seven bases only.

Interestingly, one true-positive variant was missed when applying no adapter trimming. The allele frequency of the heterozygous variant dropped from 54 to 3 % due to incorrect mapping of untrimmed adapter remains at the same genomic position (see Additional file 1: Figure S2 for details).

Table 4 shows the single-thread processing time and memory usage benchmark. The time benchmark clearly demonstrates that adapter trimming must not slow down the overall processing. Fast adapter trimmers with a good trimming performance can speed up the overall analysis time because mapping is considerably faster after trimming. These mapping times are specific for BWA. However, when using a more sophisticated mapper, e.g. Stampy [9], or an additional indel-realigner, e.g. ABRA [11], an even greater benefit of adapter trimming can be expected. A second observation from the time benchmarks is that AdapterRemoval, SeqPrep and FlexBar are probably too slow for routine application in a high-throughput setting. The peak memory usage of most tools is below 30 MB. Only PEAT and Trimmomatic use much more memory, but the reason for this remains unclear.

**Table 4:** Resources benchmark results on real data

	Trimming time	Mapping time	Variant calling time	Memory usage
no trimming	n/a	**303**	69	n/a
SeqPurge 0.1-270	38	182	65	28.8
AdapterRemoval 1.5.4	**403**	198	66	12.2
Flexbar 2.5	**165**	204	65	13.3
PEAT 1.2.2	43	241	73	**672.7**
SeqPrep 1.2	**304**	180	65	6.2
Skewer 0.1.123	24	200	66	5.7
Trimmomatic 0.32	58	177	66	**220.9**

Benchmark results of single-thread processing times (in seconds) and peak memory usage (in MB). The most notable entries are highlighted (bold)

The adapter trimming benchmarks presented so far, did not take trimming of low-quality bases into account. We excluded this feature, because not all tools in the comparison support it. Those tools that support it use basically the same approach with only minimal differences: low-quality bases are removed from the end of the read until a given base quality cutoff is exceeded. Thus, no significant performance differences between tools can be expected.

It is however interesting if quality trimming improves mapping and variant calling in general. Our benchmarks using SeqPurge and Skewer (see Additional file 1: Table S1 for details) indicate that trimming low-quality bases improves the properly-paired read count and the undertrimmed bases count. Interestingly, most of the performance improvement was already achieved at moderate quality score cutoffs (Q5 to Q15) and increasing the cutoff did not improve the performance any further. A more extensive evaluation of read trimming effects on NGS data can be found in [4].

### Benchmark results on simulated data

In our benchmark on real data we measured the adapter trimming performance using a comprehensive set of metrics. However, on real data we could not measure the tolerance towards sequencing errors. In practice, this is an important feature because it is not possible to adjust algorithm parameters for each dataset in a high-throughput setting. Thus, we benchmarked the error tolerance on simulated data with different error rates.

Table 5 shows the trimming performance on simulated data without sequencing errors. In terms of trimming time, the results are similar to the benchmark on real data: AdapterRemoval, SeqPrep and Flexbar are slower than the other tools. Likewise, Flexbar, PEAT and Trimmomatic show a lower sensitivity/specificity. SeqPurge shows a slight tendency to over-trim up to 10 bases from the end of reads that consist of simple repeats. However, this affects less than 0.02 % of the reads, which is acceptable since the focus of the algorithm lies on sensitivity.

**Table 5:** Trimming benchmark results on simulated data without errors

	Time [s]	Bases overtrimmed	Bases undertrimmed
SeqPurge 0.1-270	224	14488	0
AdapterRemoval 1.5.4	**1712**	434	0
Flexbar 2.5	**837**	**2842491**	**221979**
PEAT 1.2.2	342	**2701634**	**70617998**
SeqPrep 1.2	**1158**	1848	0
Skewer 0.1.123	185	16	0
Trimmomatic 0.32	348	0	**2225766**

Benchmark results on simulated data (5 million read pairs of 100 bp) without sequencing errors. The most notable entries are highlighted (bold)

With increasing error rate the execution time and the number of overtrimmed bases (i.e. the specificity) stayed constant for all tools, only the number of undertrimmed bases (i.e. the sensitivity) changed significantly. Thus, we now focus on undertrimmed bases of those tools that performed well on error-free data (see Additional file 1: Table S2 for the complete benchmark results). Table 6 shows the undertrimmed base counts for different error rates. These results show that SeqPrep and Skewer do not cope well with higher sequencing error rates. AdapterRemoval performs well up to 2 % error rate. Only SeqPurge performs well up to 4 % error rate, which corresponds to a very bad read quality that would not be tolerated in production data.

**Table 6:** Undertrimmed base counts on simulated data

Error rate	SeqPurge	AdapterRemoval	SeqPrep	Skewer
0.00 %	0	0	0	0
0.50 %	0	0	**866762**	**2927032**
1.00 %	0	212	**2029956**	**9321200**
2.00 %	122	48190	**3367375**	**36206230**
4.00 %	4312	**6233300**	**4248922**	**109562658**

Undertrimmed base counts on simulated data (5 million read pairs of 100 bp) with different rates of sequencing errors. The most notable entries are highlighted (bold)

## Conclusion

We have presented SeqPurge, a novel adapter trimmer for paired-end sequencing data that is based on a probabilistic approach. The performance of SeqPurge was compared to six other adapter trimming tools on an amplicon-based benchmark dataset and on simulated data.

Our comparison shows that SeqPurge is the only tool that offers a good performance in terms of sensitivity, specificity, speed and error tolerance: AdapterRemoval is very slow and not very sensitive. FlexBar is quite slow, not very specific and fails to trim short adapter remains. PEAT has a low sensitivity and specificity, fails to remove reads without insert and has a high memory usage. SeqPrep is very slow and removes good reads from the dataset. Skewer is not as sensitive as SeqPurge, but it is by far the fastest tool in the comparison. Using Skewer might be considered for large amounts of shotgun data where the runtime is more important than sensitivity, i.e. for whole genome data. Trimmomatic is not very sensitive, keeps short adapter remains and has a high memory usage.

For the real data benchmark we focused on amplicon-based sequencing data because the effects of incomplete adapter trimming can be demonstrated more easily on amplicon data than on shotgun data. A similar benchmark was done on shotgun data (data not shown). The results are comparable to the amplicon-based benchmark with two exceptions: (1) Spurious variant calls are rare in shotgun data because untrimmed adapters are generally not placed at the same genomic position; (2) adapter dimers without insert occur less frequently in shotgun data than in amplicon data.

When considering the overall runtime and file I/O load of an analysis pipeline, processing the FASTQ raw data is one of the main contributors. Thus, it is advantageous if only one tool is needed for raw read processing. SeqPurge not only offers adapter trimming functionality, but also trimming by base quality and no-call stretches. Additionally, it can merge input FASTQ files from several runs or lanes during the trimming process, and it can calculate basic quality control metrics on raw read data. The combination of these features reduces the number of passes needed to perform the basic data preparation steps significantly. To our knowledge, SeqPurge is the only adapter trimmer that combines all these features into a single tool. SeqPurge currently does not support merging of overlapping read pairs into longer single-end reads, which is supported by SeqPrep and AdapterRemoval. A detailed feature overview of other adapter trimmers from our comparison can be found in [5].

### Availability and requirements

SeqPurge is implemented in C++ and runs both under Linux and Windows. It is available under the ‘GNU General Public License version 2’ as part of the ngs-bits project: https://github.com/imgag/ngs-bits.

## Ethics approval and consent to participate

Not applicable.

## Consent for publication

Not applicable.

## Availability of data and materials

The real NGS dataset supporting the conclusions of this article is available in the European Nucleotide Archive, ftp://ftp.sra.ebi.ac.uk/vol1/ERA494/ERA494451/. The simulated data supporting the conclusions of this article was created using PERsim which is available at https://github.com/imgag/ngs-bits.

## Additional file

Additional file 1: Figure S1. Mapping of reads without insert. **Figure S2.** Variant that is suppressed by adapter contamination. **Figure S3.** High-quality reads removed by SeqPrep (example 1). **Figure S4.** High-quality reads removed by SeqPrep (example 2). **Table S1.** Benchmark results with low-quality trimming. **Table S2.** Detailed benchmark results on simulated data. (DOCX 184 kb)
