# Supplementary material for: SeqPurge: highly-sensitive adapter trimming for paired-end NGS data
Source: BMC Bioinformatics. 2016 May 10;17:208. doi: 10.1186/s12859-016-1069-7 (PMC4862148; doi:10.1186/s12859-016-1069-7)
Supplement: Additional file 1: Figure S1. — Mapping of reads without insert. Figure S2. Variant that is suppressed by adapter contamination. Figure S3. High-quality reads removed by SeqPrep (example 1). Figure S4. High-quality reads removed by SeqPrep (example 2). Table S1. Benchmark results with low-quality trimming. Table S2. Detailed benchmark results on simulated data. (DOCX 184 kb) [file 12859_2016_1069_MOESM1_ESM.docx]

### Supp. Fig. 1. Mapping of reads without insert.

This IGV screenshot shows two read pairs that have no insert, i.e. only the sequencing adapters and the following non-insert bases were sequenced. After soft-clipping start and end of the reads to fragments of 18 to 24 bases length, BWA can find a position in the reference genome where the reads can be mapped as a proper pair with a mapping quality above 30.


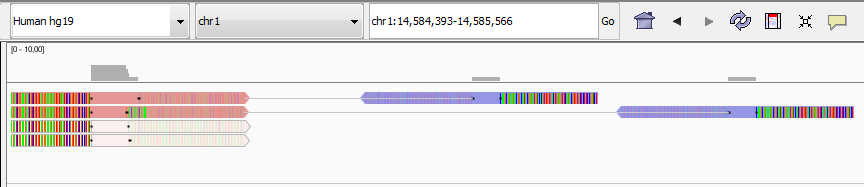


### Supp. Fig. 2. Variant that is suppressed by adapter contamination.

This IGV screenshot shows a variant that is not called because of untrimmed adapter residues. These adapter residues are not soft-clipped completely by BWA because of the first two adapter bases that match the reference genome.

### Supp. Fig. 3. High-quality reads removed by SeqPrep (example 1).


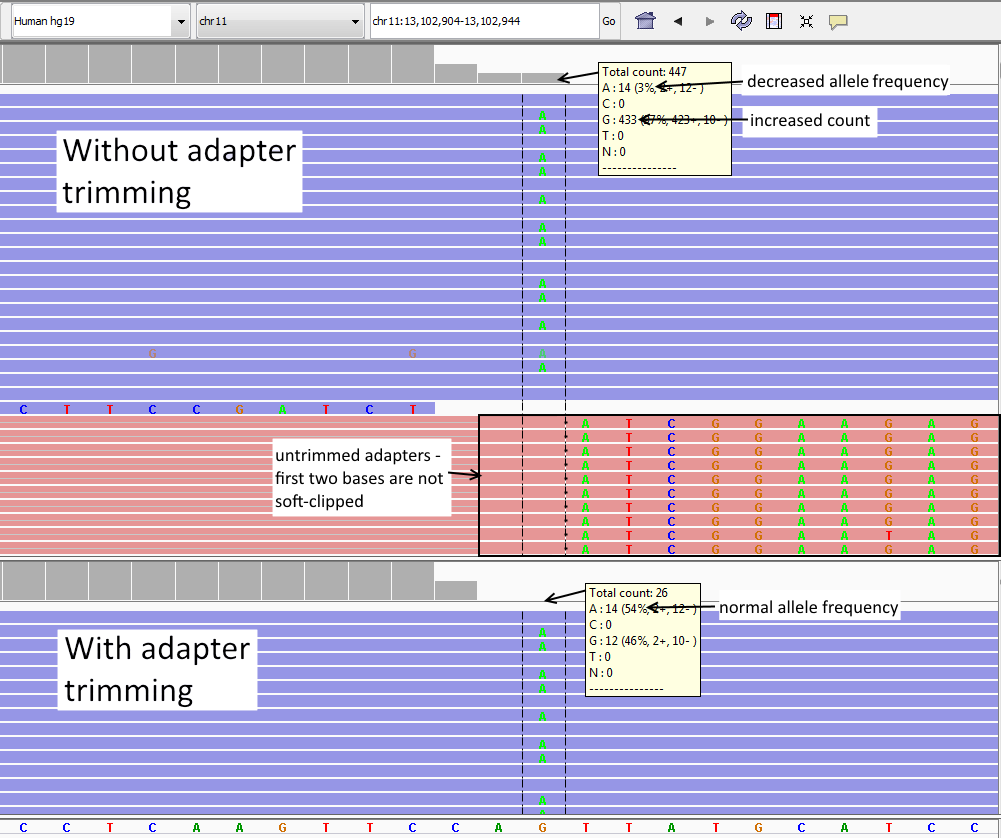


This IGV screenshot shows a region where SeqPrep removes high-quality reads that can be mapped as proper pairs.


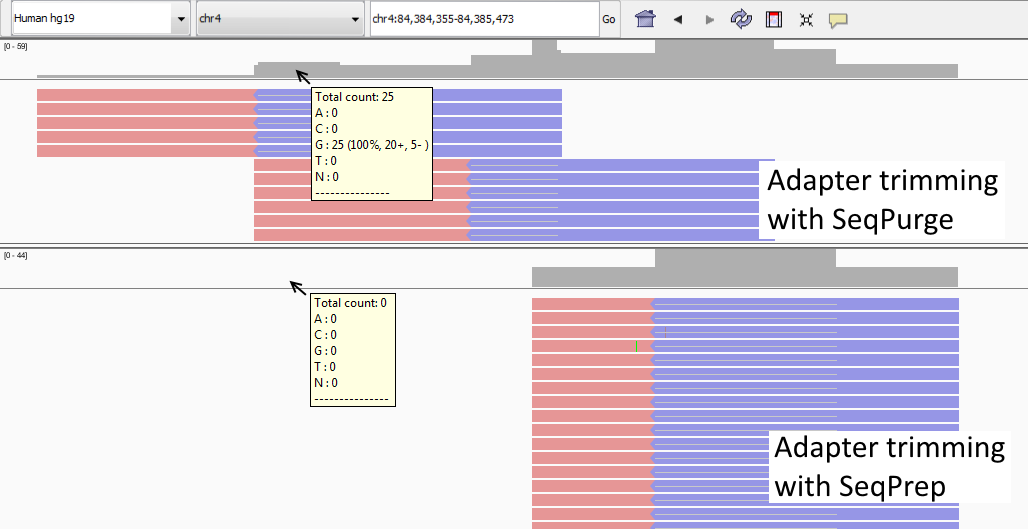


### Supp. Fig. 4. High-quality reads removed by SeqPrep (example 2).

This IGV screenshot shows a region where SeqPrep removes high-quality reads that can be mapped as proper pairs

### Supp. Table 1. Benchmark results with low-quality trimming.


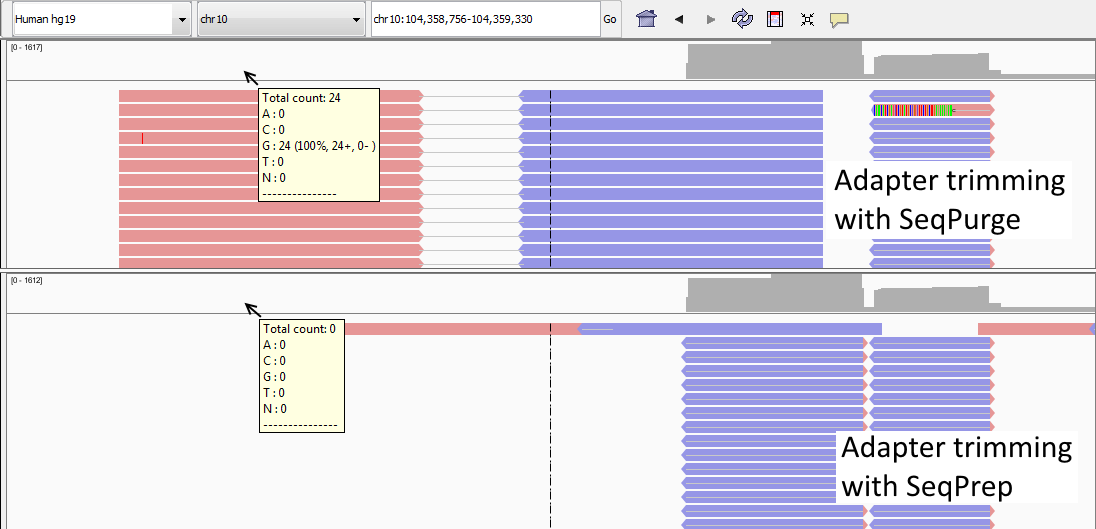


Benchmark results on amplicon dataset with both adapter and quality trimming enabled. Overtrimmed bases are not shown because adapter trimming and quality trimming cannot be distinguished.

|  | **trimming** | |  | | **mapping** | |  | | **variant calling** | |  |
| --- | --- | --- | --- | --- | --- | --- | --- | --- | --- | --- | --- |
|  | **time [s]** | **bases left** | | **adapters left** | **time [s]** | **reads  paired** | | **bases undertrimmed** | **time [s]** | **variants** | |
| SeqPurge (no quality trimming) | 39 | 142570398 | | 0 | 156 | 1021315 | | 33650 | 63 | 156 | |
| SeqPurge -qcut 5 -ncut 7 | 42 | 141810761 | | 0 | 151 | 1021864 | | 13899 | 65 | 156 | |
| SeqPurge -qcut 10 -ncut 7 | 43 | 141810761 | | 0 | 175 | 1021864 | | 13899 | 64 | 156 | |
| SeqPurge -qcut 15 -ncut 7 | 41 | 141805175 | | 0 | 169 | 1021859 | | 13899 | 65 | 156 | |
| SeqPurge -qcut 20 -ncut 7 | 45 | 141758451 | | 0 | 164 | 1021840 | | 10142 | 63 | 156 | |
| SeqPurge -qcut 25 -ncut 7 | 46 | 141641747 | | 0 | 180 | 1021763 | | 8586 | 67 | 156 | |
| SeqPurge -qcut 30 -ncut 7 | 41 | 141641747 | | 0 | 173 | 1021763 | | 8586 | 68 | 156 | |
| Skewer (no quality trimming) | 33 | 142617098 | | 425 | 166 | 1021466 | | 76800 | 64 | 157 | |
| Skewer -n -q 5 | 37 | 141939668 | | 240 | 170 | 1022095 | | 29764 | 63 | 157 | |
| Skewer -n -q 10 | 36 | 141939668 | | 240 | 169 | 1022095 | | 29764 | 65 | 157 | |
| Skewer -n -q 15 | 35 | 141936070 | | 240 | 173 | 1022091 | | 29764 | 63 | 157 | |
| Skewer -n -q 20 | 35 | 141906124 | | 240 | 163 | 1022081 | | 22081 | 64 | 157 | |
| Skewer -n -q 25 | 33 | 141904317 | | 240 | 169 | 1022079 | | 22069 | 65 | 157 | |
| Skewer -n -q 30 | 32 | 141880759 | | 240 | 157 | 1022075 | | 19100 | 65 | 157 | |

### Supp. Table 2.  Detailed benchmark results on simulated data.

Benchmark results with different error rates on 5 million simulated read pairs of 100 bp length from the coding region (CCDS).

| **0% error** | **time [s]** | **bases overtrimmed** | **bases undertrimmed** |
| --- | --- | --- | --- |
| SeqPurge 0.1-270 | 224 | 14488 | 0 |
| AdapterRemoval 1.5.4 | 1712 | 434 | 0 |
| Flexbar 2.5 | 837 | **2842491** | **221979** |
| PEAT 1.2.2 | 342 | **2701634** | **70617998** |
| SeqPrep 1.2 | 1158 | 1848 | 0 |
| Skewer 0.1.123 | 185 | 16 | 0 |
| Trimmomatic 0.32 | 348 | 0 | **2225766** |
| **0.5% error** | **time [s]** | **bases overtrimmed** | **bases undertrimmed** |
| SeqPurge 0.1-270 | 213 | 14382 | 0 |
| AdapterRemoval 1.5.4 | 1704 | 362 | 0 |
| Flexbar 2.5 | 796 | **2846259** | **228660** |
| PEAT 1.2.2 | 347 | **2642448** | **70906774** |
| SeqPrep 1.2 | 1128 | 1346 | **866762** |
| Skewer 0.1.123 | 183 | 22 | **2927032** |
| Trimmomatic 0.32 | 440 | 0 | **2247700** |
| **1% error** | **time [s]** | **bases overtrimmed** | **bases undertrimmed** |
| SeqPurge 0.1-270 | 240 | 17344 | 0 |
| AdapterRemoval 1.5.4 | 1654 | 390 | 212 |
| Flexbar 2.5 | 822 | **2850863** | **232029** |
| PEAT 1.2.2 | 369 | **2553174** | **70768624** |
| SeqPrep 1.2 | 1072 | 1664 | **2029956** |
| Skewer 0.1.123 | 214 | 12 | **9321200** |
| Trimmomatic 0.32 | 439 | 0 | **2391308** |
| **2% error** | **time [s]** | **bases overtrimmed** | **bases undertrimmed** |
| SeqPurge 0.1-270 | 240 | 13774 | 122 |
| AdapterRemoval 1.5.4 | 1734 | 158 | 48190 |
| Flexbar 2.5 | 838 | **2857281** | **243509** |
| PEAT 1.2.2 | 370 | **2455562** | **70795822** |
| SeqPrep 1.2 | 1085 | 1390 | **3367375** |
| Skewer 0.1.123 | 179 | 0 | **36206230** |
| Trimmomatic 0.32 | 474 | 0 | **4334954** |
| **4% error** | **time [s]** | **bases overtrimmed** | **bases undertrimmed** |
| SeqPurge 0.1-270 | 226 | 10978 | 4312 |
| AdapterRemoval 1.5.4 | 1745 | 32 | **6233300** |
| Flexbar 2.5 | 892 | **2857273** | **284203** |
| PEAT 1.2.2 | 416 | **2224498** | **70818288** |
| SeqPrep 1.2 | 1141 | 1014 | **4248922** |
| Skewer 0.1.123 | 190 | 0 | **109562658** |
| Trimmomatic 0.32 | 460 | 0 | **25750498** |
